# On the Use of Nitric Acid as an Anti-Periodic

**Published:** 1854-11

**Authors:** George Mendenhall

**Affiliations:** Professor of Obstetrics, &c., in the Miami Medical College of Cincinnati


### Record of Medical Science.

On the use of Nitric Acid as an Anti-Periodic. By George Mendenhall, M. D., Professor of Obstetrics, &c., in the Miami Medical College of Cincinnati.—My attention has recently been called to the use of Nitric Acid in the treatment of intermittent fever; and as a substitute for quinine in this class of disease is a matter of some consequence on many accounts, I am induced to lay it before your readers that it may more fully undergo the ordeal of experience, and its true value be tested by different observers.

The facts upon which this paper is based, are taken mainly from an Inaugural Dissertation, presented to the Trustees and Faculty of the Miami Medical College, for the degree of Doctor of Medicine, by E. T. Bailey, M. D., of Emmetsville, Indiana.

He states, that in the section of country in which he resides, there is a large portion of marshy land, and therefore the circumstances are favorable to the development of autumnal fevers. His attention was first attached to the use of nitric acid in the treatment of intermtttent fevers, by noticing its effects in a case of chronic intermittent, which was attended with profuse night sweats, and for which complication he administered the remedy. In this case, there had been daily paroxysms for the preceding five days; night-sweats profuse, the tongue coated, and the bowels constipated. Nitric acid was given in doses of six drops, diluted with water, in the evening; and he was agreeably surprised to find that the paroxysms did not return on the following day; and this circumstance induced him to try its effects in other cases as an anti-periodic.

Since that time he has treated over ninety cases of intermittent fever with this article, with remarkable success. Of this number, all recovered promptly except ten, and in every one of these unsuccessful cases, the remedy was discontinued contrary to directions.

Fifteen of the whole number were of the tertian type, and seventy-five of the quotidian. In fifty cases, there was no return of the chill after commencing the use of the acid. The others were rarely attended by more than one paroxysm, and in no case by a third. When the patient had a paroxysm after taking the medicine, it was in every case diminished in intensity and duration.

In Dr. Bayley's practice, this remedy has entirely superseded every other article for the purpose of interrupting the paroxysms of intermittents. His mode of proceeding is to give from five to eight drops of the commercial nitric acid, properly diluted, once in six hours, without regard to intermissions or exacerbations. Cathartics and alterants may be necessary for the purpose of changing certain conditions of the system; but so far as the interruption of the paroxysms is concerned, the acid may be given without any preparation of the system whatever, if we choose to do so.

The following cases are selected from among many others, in order to show the different varieties of the disease, and the mode of treatment pursued:

---

Case I.—Mr. L. S., aged 55, of temperate habits, called upon him on the 12th of July, 1852. In May previous, he was attacked with intermittent fever, which was cured by quinine, but relapsed in six weeks, since which, the disease has continued with daily paroxysms. The tongue was furred, and the bowels constipated. A purgative of calomel and rhubarb was ordered at bed time. A violent fever followed the chill, which occurred at 11 o'clock, A. M., of this day. Five drops of nitric acid was directed to be taken every six hours, diluted so as to make a pleasant drink, to be commenced on the following morning, the 13th. The calomel and rhubarb produced two copious operations from the bowels, prior to commencing the nitric acid. Three hours after the exhibition of the remedy at 6 o'clock, as directed, a chill came on, followed by a high fever. The medicine was continued through the day.

14th. The patient is free from a paroxysm, no fever; the tongue clean and moist; and the medicine was directed to be continued as before.

15th. The tongue improving; bowels regular, and pulse very little excited; no chill. Medicine continued.

16th. Patient has entirely recovered.

Forty-five drops only were taken in this case, as the medicine was not given at night.

---

Case II.—Sept. 10th, 1852, was called to see Master L. A., aged six years. He has had intermittent fever since the 6th inst., with daily paroxyms, followed by high fever. The tongue was moist, and the bowels open. Four drops of nitric acid were ordered, which was diluted as before, so as to be easily taken; one dose to be taken at 12 o'clock, and the second at 6 in the evening; to be resumed at 6 the following morning, and repeated at 12 o'clock.

11th. Visited the patient in the afternoon. He had not had any chill; the tongue was clean and skin moist, with no excitement of the pulse. Discharged cured.

In this case, the medicine was discontinued to see whether the effect would be permanent.

12th. No return of the paroxysm. Sixteen drops were taken in this case.

---

Case III.—Sept 20th, 1853. Saw Miss D. N., aged 28. Three days ago, she was attacked with intermittent fever, the paroxysms of which commenced at 10 o'clock every day, and the chill was followed by high fever. A mild purgative was ordered at bed time; and sixty drops of nitric acid were diluted with two ounces of water, and directed to be taken in nine doses, at the rate of three doses per day, commencing on the morning of the 21st.

25th. Saw the patient to-day, and found that there had been no return of the paroxysms for three days. In this case, sixty drops were taken.

Several other cases are reported with the same result, but it is not necessary to repeat them here.

It is stated that relapses seldom occur—much less frequently than after the use of quinine, but upon this point more precision is required. It has been administered in all the various forms of intermittent fever which were presented to the author of the essay, and no unpleasant effects observed in any case. Should they be verified by the experience of others, this remedy will be found to be a valuable agent, and to possess the following advantages:

*First.* Certainly as an Anti-Periodic. *Second.* The power of invigorating the general system, while at the same time it has alterant properties. *Third.* Facility of exhibition, being much easier taken than quinine, barks, &c. *Fourth.* Freedom from the unpleasant effects which sometimes follow these remedies. *Fifth.* Cheapness of the article, which is often of considerable importance in miasmatic regions.—*West. Lancet.*

---

On Constipation. By Professor Trousseau.—Constipation should be defined, a difficulty in the execution of the fœcal matters, independent of any organic lesion. We should not rank among the constipated those in whom defecation is impeded by a tumor or hernia, or invagination, or other mechanical obstacle. Simple constipation is nothing more than absence of alvine evacuations.

Such a condition may depend on many causes. First, there is a sort of phisiological constipation, connected with the constitution of the patient; an individual eats but little, and is endowed with great powers of assimilation. The quantity of excrementitious matters, in such a case, are insufficient to stimulate the in

